# Research Progress on the Mechanism and Function of Histone Acetylation Regulating the Interaction between Pathogenic Fungi and Plant Hosts

**DOI:** 10.3390/jof10080522

**Published:** 2024-07-26

**Authors:** Xiaokang Zhang, Yuzhu Zhou, Yangzhi Liu, Boqiang Li, Shiping Tian, Zhanquan Zhang

**Affiliations:** 1 Key Laboratory of Plant Resources, Institute of Botany, Chinese Academy of Sciences, Beijing 100093, China; zhangxk@ibcas.ac.cn (X.Z.); zhouyz@ibcas.ac.cn (Y.Z.); liuyz@ibcas.ac.cn (Y.L.); bqli@ibcas.ac.cn (B.L.); tsp@ibcas.ac.cn (S.T.); 2 University of Chinese Academy of Sciences, Beijing 100049, China; 3 State Key Laboratory of Vegetable Biobreeding, Institute of Vegetables and Flowers, Chinese Academy of Agricultural Sciences, Beijing 100081, China

**Keywords:** histone acetylation, histone acetylation enzymes, histone deacetylation enzymes, fungal pathogenicity, disease resistance

## Abstract

Histone acetylation is a crucial epigenetic modification, one that holds the key to regulating gene expression by meticulously modulating the conformation of chromatin. Most histone acetylation enzymes (HATs) and deacetylation enzymes (HDACs) in fungi were originally discovered in yeast. The functions and mechanisms of HATs and HDACs in yeast that have been documented offer us an excellent entry point for gaining insights into these two types of enzymes. In the interaction between plants and pathogenic fungi, histone acetylation assumes a critical role, governing fungal pathogenicity and plant immunity. This review paper delves deep into the recent advancements in understanding how histone acetylation shapes the interaction between plants and fungi. It explores how this epigenetic modification influences the intricate balance of power between these two kingdoms of life, highlighting the intricate network of interactions and the subtle shifts in these interactions that can lead to either mutual coexistence or hostile confrontation.

## 1. Introduction

**Figure 1:**
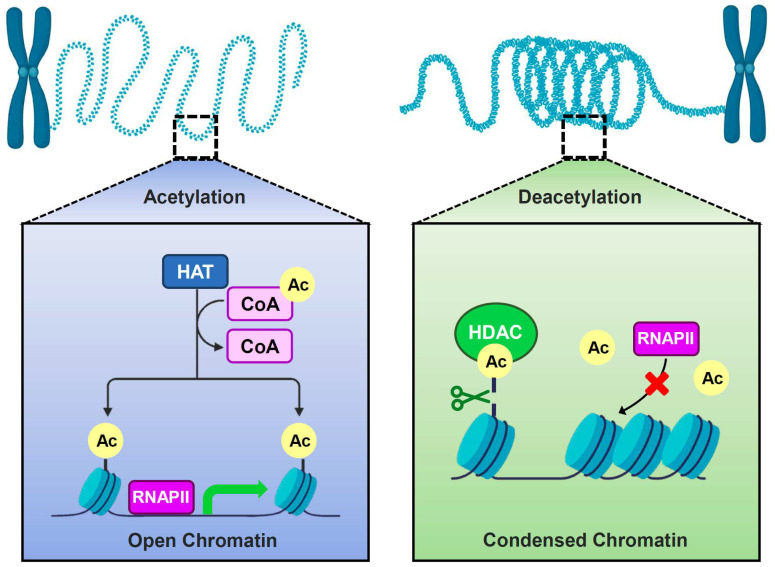
Schematic diagram of histone acetylation modification. Histone acetylation modification regulates gene expression by depositing acetylation marks on chromatin. HATs (histone acetyltransferases) utilize acetyl-CoA as a donor to acetylate histones, causing chromatin to open, and allowing RNA polymerase II to bind to the promoter to induce target gene expression. Conversely, HDACs (histone deacetyltransferases) recognize histone acetylation marks and remove acetylation modifications, leading to chromatin condensation and inhibiting transcription by preventing RNA Polymerase II binding.

Histone modifications that occur post-translationally are pivotal in governing the intricate process of transcription. These alterations play a fundamental role in modulating the expression of genes, thereby influencing a wide range of biological processes [1]. These modifications encompass methylation, acetylation, phosphorylation, sulfonylation, and ubiquitination [2]. Acetylation, a reversible and dynamic process, is regulated by histone acetyltransferases (HATs) and histone deacetylases (HDACs), which have opposing functions and engage in intricate interactions [3]. HAT enzymes mediate the transfer of acetyl groups from acetyl-CoA to precise lysine residues within histone amino terminal tails, thereby modifying the chromatin configuration and ultimately activating transcription. Conversely, HDACs reverse this process by catalyzing the removal of acetyl groups, resulting in the suppression of transcriptional activity [4]. These enzymes govern the acetylation state of histone, essential for chromatin restructuring and transcriptional governance (Figure 1) [5,6].

The interaction between plants and fungi is a complex and intricate process involving numerous regulatory mechanisms. Recent research has revealed the significant role of histone acetylation in the plant–fungi interaction.

In addition to histone acetylation, the interaction between plants and fungi also involves various other epigenetic modifications, including DNA methylation, histone methylation, phosphorylation, ubiquitination, and succinylation [7]. Moreover, recent studies have shown that histone acetylation, in conjunction with other epigenetic modifications, forms a sophisticated regulatory network that governs plant immunity and fungal virulence [8,9]. Understanding histone acetylation and its interaction with other epigenetic modifications not only sheds light on fundamental aspects of plant–pathogen interactions but also holds promise for developing novel strategies to enhance crop resistance against devastating fungal diseases.

This review synthesizes the current knowledge and recent advancements in the field, offering a comprehensive perspective on the significance of histone acetylation in the interplay between pathogenic fungi and their plant hosts. A thorough investigation into the regulatory mechanisms of histone acetylation is significant for uncovering the essence of interactions between plants and pathogenic fungi, promising a brighter future for sustainable crop protection and food security.

## 2. Enzymes for Histone Acetylation Modification

Yeast is an important model organism for studying histone acetylation. Most histone acetylation enzymes and deacetylation enzymes in fungi were originally discovered in yeast, serving as the initial point of exploration for their functions and mechanisms. Subsequently, homologous proteins to those found in yeast were also identified in other fungal species. Scientists categorize histone acetylation-related proteins based on their functions discovered in yeast [10]. Based on the study of yeast, the mechanisms of histone acetylation in gene expression regulation, cell cycle, DNA damage repair, and other aspects were revealed [11]. These studies provide an important foundation for us to understand the biological role of histone acetylation in gene expression regulation in humans and other organisms. In the following section, we initially provide a summary of histone acetylation enzymes and deacetylation enzymes in yeast.

### 2.1. The Biological Function of Histone Acetyltransferases in Yeast

Histone acetyltransferases orchestrate the acetylation process of lysine residues positioned within histone proteins’ N-terminal tails and globular domains, employing acetyl-CoA as the acetyl-donating agent. This acetylation process converts chromatin into a transcriptionally permissive configuration, thereby promoting gene expression [10,11,12,13]. Relying on preserved structural patterns, the classification of HATs encompasses five distinct families: GNAT (GCN5-related N-acetyltransferases), MYST (MOZ, YBF2/SAS3, SAS2, TIP60), p300/CBP (CREB-binding proteins), basal transcription factors, and nuclear receptor co-activators [11,12,13,14].

The GNAT family is highly similar to the enzyme GCN5 (general control non-depressible 5) in yeast, which usually represents the GNAT family and has been extensively studied. GCN5 plays a role in the overarching governance of signaling pathways that regulate amino acid synthesis and display transcription-related histone acetyltransferases [15,16,17]. Its activity depends on associations in diverse multisubunit assemblies, such as SAGA (SPT-ADA-GCN5-acetyltransferase) and ADA (alteration/deficiency in activation 2) [18]. The SAGA complex, composed of the histone acetyltransferase GCN5 and its interacting partners, ADA2 and ADA3, serves as a multimodule complex assembly regulating eukaryotic transcription through modulation of histone acetylation [19,20]. GCN5 holds a critical position as the first HAT identified to target the N-terminal lysine residues of histones H3 and H2B [17,21,22]. This enzyme is found in intact SAGA protein complexes, alongside ADA2 and ADA3, which associate with both free histones and nucleosomal histones, exhibiting robust HAT activity [18,23,24]. Specific *ada2* mutations in yeast result in the dissociation of the GCN5 subunit from the SAGA complex, significantly impacting cell growth and transcription in vivo [25,26,27].

The acetyltransferase MYST protein family is highly conserved from yeast to humans, whose histone acetylation activities play important roles in diverse nuclear processes, including transcription, DNA repair, and DNA duplication [28]. In *Saccharomyces cerevisiae*, MYST family proteins contain three members: ESA1 (essential SAS2-related acetyltransferase), SAS2 (something about silencing 2), and SAS3 (something about silencing 3). ESA1 mainly acetylates lysines 5, 8, 12, and 14 of histone H4 [29,30]. As a founding member of the MYST family, SAS2 serves as a catalytic subunit of the SAS complex [31,32]. In *S. cerevisiae*, SAS2 associates with SAS4 and SAS5 to form the SAS complex, which is essential for SAS2 HAT activity [32,33]. Furthermore, SAS2 interacts with CAC1 (chloroplastic acetylcoenzyme A carboxylase 1), which is the primary subunit of the chromatin assembly factor I, as well as the nucleosome assembly factor ASF1 (anti-silencing factor 1). Deletion of either CAC1, ASF1, or SAS2 results in similar impacts on gene silencing. Previous studies have demonstrated that SAS2 catalyzes the acetylation of H4K16 [32]. This acetylation of lysine residues on histone H4 is known to loosen the chromatin structure, facilitating the interaction of transcription factors and coactivators with DNA [34,35]. In yeast, the HAT activity of SAS2 leads to high levels of H4K16ac, which inhibits telomere heterochromatin formation by impeding the function of SIR, a protein associated with telomere gene silencing [36]. MST1 is an essential protein required for chromosome segregation and DNA damage responses [37], and MST2, another MYST member homologous to SAS2 function, is involved in telomere silencing in *Schizosaccharomyces pombe* [38]. SAS3-mediated acetylation of H3K14 is necessary for transcriptional extension [39], while SAS3, the yeast MYST protein with the least amount of research, is a catalytic component of the NuA3 (nucleosomal acetyltransferase of histone H3) histone acetyltransferase complex [40]. Concurrent disruption of SAS3 and GCN5 leads to widespread loss of H3 acetylation and cell cycle stalling in the G2/M phase [41].

RTT109 (regulator of Ty1 transposition gene product 109) belongs to the fungus-specific KAT11 family of histone acetyltransferases, which is mainly responsible for the acetylation of H3K5 [42]. It is necessary for the assembly of DNA replication-coupled nucleosomes and genome stability [43]. The activation of RTT109 occurs via an unidentified mechanism, involving the attachment of two unique histone chaperones, VPS75 (vacuolar protein sorting-associated protein 75) and ASF1 [44], and the acetylation of H3K56 is eliminated and DNA repair is hampered in yeast cells when *RTT109* or *ASF1* is absent [45,46].

### 2.2. The Biological Function of Histone Deacetyltransferases in Yeast

Histone deacetylases form an enzymatic class that facilitates the removal of acetyl groups from the ε-amino group of lysine residues situated at the N-terminal extremities of histones. This enzymatic reaction, termed deacetylation, effectively reinstates the positive charge to the histone tails, thereby regulating their functional properties [47]. Unlike histone acetylation, deacetylation prompts a tighter binding of histones to DNA, leading to the formation of condensed chromatin structures (heterochromatin), which renders the DNA inaccessible for transcription processes. HDACs frequently occur in extensive multi-protein assemblies, alongside transcriptional co-suppressors, further implicating them in transcriptional inhibition [11]. HDACs are divided into four groups based on sequence homology and evolutionary study [48].

Class I HDACs share homology with RPD3 (reduced potassium dependency 3) in yeast, encompassing HDAC1-3 and HDAC8. RPD3 is an important member of Class I HDAC in *S. cerevisiae*, exhibiting the capability to deacetylate histone H3 and H4 [49]. In yeast, RPD3 emerges as a crucial co-factor within the regulatory network, modulating gene expression in response to environmental stressors [50]. The RPD3 protein serves in two distinct molecular complexes. One of these, the smaller RPD3S complex, comprises five subunits: RPD3, SIN3, UME1, RCO1, and EAF3, and possesses a total molecular weight of 400 kDa. This complex operates subsequently to the histone methyltransferase, SET2 [51,52,53]. The recruitment of RPD3S to open reading frames is facilitated by histone H3 lysine 36 methylation, where it subsequently removes acetyl groups from acetylated lysines on histones H3 and H4, thus maintaining a hypoacetylated state in these genomic regions. The SET2–RPD3S signaling pathway is crucial for suppressing cryptic transcription, a phenomenon that frequently occurs within open reading frames [51,52,53]. RPD3S exhibits a preferential affinity for binding to a dinucleosome, thereby enhancing the deacetylation process of its constituent two mononucleosomes [54,55,56]. On the contrary, the larger complex (RPD3L) is recruited to promoters via interactions with specific DNA-binding proteins, fulfilling a pivotal role in transcription repression [57,58]. HOS2 (Hda one similar 2) is another member of Class I HDACs and is a constituent of the SET3C (Su(var)3–9, enhancer-of-zeste and trithorax 3 complex) [59]. HOS2 specifically removes acetyl groups from H3 and H4 lysines, functioning antagonistically against Esa1 (a kind of HAT of the MYST family) in the DNA damage response [60,61]. Hos2 stands out as an indispensable factor for gene activation in *S. cerevisiae*, distinguishing it from the other Class I HDACs [61].

Class II HDACs exhibit homology to HDA1 (histone deacetylase 1) in yeast, encompassing HDAC4-7 and HDAC9-10. HDA complex, a specific Class II HDAC complex, comprises three distinct subunits: the catalytic HDA1, alongside the regulatory cofactors HDA2 and HDA3. This complex precisely targets acetylated lysines on histone H3 (positions K9, K14, K18, K23, and K27) and histone H2B (positions K11 and K16) for deacetylation [62] and inhibits and rivals GCN5 for occupancy on the promoters [63]. Disruption of HDA results in augmented H3K18 acetylation on promoters and consequently enhances transcriptional activation in the trehalose metabolic pathway. This upregulation confers resilience to DNA damage and osmotic stresses, ultimately contributing to the extended lifespan of yeast [64]. Both Class I and Class II HDACs rely on Zn^2+^ as a cofactor to facilitate their deacetylase function.

Class III HDACs form a distinct category of histone deacetylases, whose catalytic function relies on the presence of NAD^+^ [65,66,67,68]. The inaugural member of this sirtuin class, denominated as SIR2 (silent information regulator 2) originating from *S. cerevisiae*, interfaces with additional SIR proteins and facilitates the suppression of heterochromatin-analogous regions in this yeast species via the deacetylation process targeted at the H4 lysine 16 residue (H4K16) [36,69].

Despite the extensive research conducted on Class I, II, and III HDACs in fungi, Class IV HDACs constitute a unique group, comprising solely HDAC11. This enzyme, HDAC11, exhibits remarkable conservation and is found across all eukaryotes, fungi being the sole exception [70].

## 3. Effect of Histone Acetylation on Fungal Pathogenicity

In the context of plant–fungus interaction, histone acetylation modulates the outcome by regulating the expression of plant immune-related genes and fungal pathogenicity-associated genes. Regarding fungi, histone acetylation exerts a significant impact on their pathogenicity by modulating pathogenicity-related gene expression, thereby influencing their infectivity and virulence on plants.

### 3.1. Histone Acetyltransferase

GCN5 plays a crucial role in regulating gene expression across multiple pathways vital for the virulence capabilities of fungi and their sensitivity to antifungal agents [71,72]. In *Aspergillus flavus*, the homolog of GCN5, designated as AflGCNE, plays a pivotal role in morphogenesis, aflatoxin biosynthesis, stress adaptation, and pathogenicity [73]. Similarly, in *Bacillus brasiliensis*, GCN5 acetylates multiple targets on histone H3, thereby contributing to the activation of genes involved in asexual development, dimorphic transition, and pathogenicity [74]. Recent research has discovered that the compound phenazine-1-carboxamide (PCN), secreted by a biological bacterium, effectively inhibits fungal virulence by directly binding to and suppressing the enzymatic activity of GCN5 in *Fusarium graminearum* [75]. Contemporary research has demonstrated that ADA2 and ADA3 play vital roles in regulating various aspects of *Beauveria bassiana*, including its growth, asexual development, stress resistance, and virulence, by modulating GCN5 enzyme activity and histone H3 acetylation, as evidenced by gene knockout and complementary experiments. These observations further underscore the influence of transcriptional adaptor proteins (ADA2 and ADA3) on GCN5’s catalytic activity, substrate specificity, and its capacity to regulate transcriptional activation via histone acetylation [47,76].

MYSTs play a pivotal role in pathogenicity and environmental adaptability among pathogenic fungi. Take SAS2 in *Candida albicans* as an example, it has the ability to acetylate diverse lysines in histone H4, and its deletion mutant exhibits heightened sensitivity to heat, genotoxicity, and oxidative stress [77]. In addition, MYSTs are critical for the synthesis of fungus secondary metabolites in *Pestalotiopsis microspore* [78]. In *Aspergillus nidulans*, elevated expression of ESA1 boosts the production of secondary metabolites via the augmentation of H4K12 acetylation [79]. More recently, the disruption of HAT1, a SAS3 homolog of *S. cerevisiae*, increased the expression of orphan secondary metabolic genes and led to a total loss of H3 acetylation in *Metarhizium robertsii* [80]. The absence of *BcSAS2* in *Botrytis cinerea* brings about a substantial diminution in histone H4 acetylation levels, particularly H4K16ac, ultimately affecting its pathogenicity and sensitivity to oxidative stress [81].

In *Aspergillus flavus*, RTT109 orchestrates a range of biological processes, including growth, the production of conidia, nuclear formation, toxin biosynthesis, the response to environmental stresses, and infestation capabilities [82]. In *A*. *flavus*, the Δ*rtt109* mutant exhibits significant impairments, rendering it incapable of producing asexual spores and suppressing sclerotia synthesis. Additionally, it downregulates the expression of genes crucial for the biosynthesis of *brlA* and *abaA*. Notably, the absence of *RTT109* in *Aspergillus fumigatus* leads to severe defects in trophic growth and conidiation, a decrease in virulence, and enhanced sensitivity to genotoxic agents [83]. Recent studies have found a notable influence of RTT109 on the transcription and expression patterns of pivotal genes pertaining to *Monascus’* development, morphogenesis, and secondary metabolism. The absence of *RTT109* notably impairs conidial production and colony expansion and increases the production of *Monascus* pigment from citrus [84].

### 3.2. Histone Deacetylase

RPD3 is not confined to yeast but is also present in filamentous fungi, where it performs specialized functions. In *B. bassiana*, this enzyme plays a pivotal role in regulating both transcriptional and posttranscriptional lysine modifications of histone associated with genes involved in central developmental pathways. The absence of RPD3 leads to profound growth deficiencies, conidial reduction, and a marked decrease in virulence [85]. DEP1, serving as a constituent of the RPD3L complex, regulates vegetative growth, ROS buildup, and disease-causing potential in *Fusarium pseudograminearum* [86]. However, in certain filamentous fungal species, including *A. nidulans*, *A. fumigatus*, *B. cinerea*, and *Magnaporthe oryzae*, the disruption of *Rpd3* is lethal. In *B. cinerea* and *M. oryzae*, the overexpression of *RPD3* leads to a significant impairment in the development of infection structures, a weakened response to oxidative stress, and a substantial decrease in virulence, thereby adversely affecting their pathogenic capabilities [87,88].

Accumulating evidence suggests that HOS2 occupies a crucial position in fungal pathogenicity. In *Cochliobolus carbonum*, HOS2 exerts a significant influence on the expression of extracellular depolymerases, thereby affecting its virulence [89]. HDF1 in *F. graminearum* is a homolog of HOS2 and participates in spore generation, deoxynivalenol synthesis, and plant infection processes. [90]. In *C. albicans*, the SET3C specifically dampens the cAMP–PKA signaling pathway, suppressing the shift from yeast form to filamentous state and regulating the switch between white and opaque phenotypes [91,92]. In *Ustilago maydis*, HOS2 functions as a crucial downstream element of the cAMP–PKA signaling cascade. Through the deacetylation of H4K16, it exerts direct regulation over the expression of mating-type genes, thereby playing an indispensable role in facilitating the dimorphic transition and the progression of pathogenic development [93]. However, in *M. oryzae*, the HOS2-deacetylated histone site is H3K18 [94]. This variation suggests that HOS2 could regulate diverse arrays of targeted genes in different fungi, connected to definite cellular roles.

In the context of regulating adaptation and virulence, HDA1 fulfills a pivotal role as a mediator, orchestrating the transcription of essential genes that are necessary for governing mating and virulence processes [95]. Multiple investigations have demonstrated HDA1’s capacity to modulate, either positively or negatively, the expression of genes responsible for secondary metabolite production in filamentous fungal pathogens [96,97,98,99]. In *Fusarium fujikuroi*, HDA1 is essential for germination, vegetative proliferation, and fungal pathogenicity [97]. In *U. maydis*, HDA1 plays a critical role in teliospore maturation and functions as a suppressor of *MIG1*, a biotrophic-associated gene, encoding a unique, secreted, cysteine-rich, hydrophilic protein that is distinctly expressed during the process of infection [100,101].

It has been established that specific Class I and II HDACs exhibit targeting capabilities towards nonhistone proteins, and RPD3, in particular, has been identified as a suppressor of autophagy, as its absence leads to an elevation in ATG3 acetylation and subsequent acceleration of autophagy in yeast [102]. Additionally, both HDA1 and RPD3 are involved in the deacetylation of HSP90 (heat shock protein 90), a crucial molecular chaperone essential for drug tolerance and the pathogenesis of *C. albicans*. This deacetylation leads to an impaired chaperone activity [103]. The antagonistic interplay between ESA1/NuA4 (nucleosomal acetyltransferase of histone H4) and HDA1 modulates the acetylation and deacetylation of EAF1 at the K173 residue. This mechanism facilitates the integration and dissociation of NuA4 and SWR1 (ATP-dependent chromatin remodeling complex) in *C. albicans*, ultimately regulating the expression of hypha-specific genes and orchestrating the transition from yeast to the hyphal growth form [104].

In filamentous fungi, this chromatin-silencing function of SIR2 is broadly observed and has been documented in *Neurospora crassa* and *A. nidulans* [105,106,107], suggesting an ancient and fundamental role in silencing. Beyond SIR2, additional sirtuins have been delineated throughout diverse organisms. All the other sirtuins have been associated with chromatin-silencing mechanisms, along with their direct involvement in gene regulation in *S. cerevisiae* and *S. pombe*, such as Hst1 to 4 in *S. cerevisiae* and HST2 and 4 in *S. pombe* [108,109,110]. Amongst the sirtuin family, SIR2 has emerged as a key regulator of diverse fungal biological processes. For example, in human pathogenic fungi *Candida glabrata*, SIR2 suppresses the expression of the EPA adhesin gene, which is critical for infection [111]. In *Cryptococcus neoformans*, SIR2 is essential for virulence, although the underlying mechanism remains to be elucidated [112]. Until now, the prime example of SIR2’s function in plant pathogens is found in *M. oryzae*. In this rice pathogen, SIR2 is presumably involved in infection by deacetylating the MoJMJC repressor, resulting in an elevation in superoxide dismutase expression and thus facilitating the detoxification of ROS [113].

Briefly, HACs and HDACs co-regulate the histone acetylation and deacetylation to influence various life processes in fungi.

## 4. Effect of Histone Acetylation on Plant Disease Resistance

**Figure 2:**
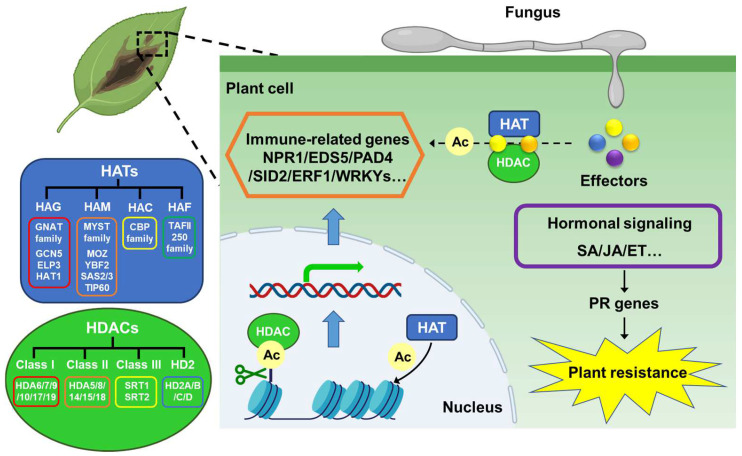
HATs and HDACs in the interaction between plant pathogenic fungi and hosts. In plants, HATs and HDACs of different families dynamically regulate histone acetylation during plant–fungi interaction. Histone acetylation regulates the transcriptional expression of immune-related genes, such as NPR1, EDS5, ERF1, WRKYs, etc., and activates downstream plant hormone signaling pathways, leading to changes in the expression of pathogenesis-related (PR) genes and plant resistance. Meanwhile, pathogenic fungi can secrete a series of effectors during the infection process, and certain effectors can competitively bind to HATs or HDACs to affect the histone acetylation process, thereby affecting the expression of immune-related genes and plant resistance.

In plants, histone acetylation occupies a central position in several levels of plant immune response. Histone acetylation can enhance disease resistance in plants by modulating the expression of genes that have a connection with disease resistance. In addition, histone acetylation can also affect plant hormone signal transduction pathways, thereby orchestrating plant growth and maturation (Figure 2).

### 4.1. Histone Acetyltransferase

Histone acetylation is very important to plant immunity. For example, the *Gossypium hirsutum*-expressing CaM7 (calmodulin 7), with mutations at the acetylation sites, is much more susceptible to *Verticillium dahliae* than those expressing wild-type CaM7 [114]. The HATs in plants are categorized into four distinct groups: GNAT family; MYST family (including MOZ, YBF2, SAS3, SAS2, and TIP60); CBP family (cAMP response-element binding protein), and TAFII250 family (factors for TATA-binding protein) [115,116]. The respective HATs, designated as HAG, HAM, HAC, and HAF [115] (Table 1), serve as crucial regulators of gene expression throughout plant development, exogenous hormonal signaling, and adaptation to environmental stresses [117,118,119,120]. The HAT gene family exhibits varying member counts among plant species, with twelve HATs identified in *Arabidopsis thaliana* [115], eight in rice [121], and thirty-two in tomato [122]. The GNAT group consists of three subfamilies: GCN5, ELP3, and HAT1-like acetyltransferases, namely HAG1, HAG2, and HAG3 [115]. GCN5 serves as a crucial catalytic subunit within various multi-protein HAT complexes, playing a pivotal role in plant development and enhancing resilience against abiotic stressors, encompassing heat, drought, cold, salt, and phosphate deficiency [116,123,124]. In *A. thaliana*, GCN5 promotes histone H3K14 acetylation to regulate the expression of salicylic acid (SA) synthesis-related genes [125]. In *Glycine max*, when infected by *Phytophthora sojae*, cytoplasmic effector PsAvh23 competitively binds to GmADA2 and dissociates the GmADA2-GmGCN5 complex, leading to the decrease in GmGCN5-mediated H3K9 acetylation levels and repression of defense-related genes [126]. In *A. thaliana*, the histone acetyltransferase ELP3, a component of the Elongator complex, positively modulates plant immunity by enhancing the expression of defense-related genes [127]. In *Triticum aestivum* L., TaHAG1 can directly interact with plant-specific zinc-binding protein TaPLATZ5 and together upregulate the expression of immune-related gene TaPAD4 by increasing H3 acetylation [128]. HAMs are the subunits of a plant’s NuA4 histone acetyltransferase complex. In *A. thaliana*, HAM1 and HAM2 mediate the acetylation of H4 and regulate chloroplast development and photosynthetic gene transcription [129]. In *Manihot esculenta*, the expression of *MeHAM1* is upregulated by cassava bacterial blight, and subsequently, MeHAM1 activates H3K9 and H4K5 acetylation to upregulate the expression of SA biosynthetic genes [130]. CBP family acetyltransferases are transcriptional coactivators that participate in many developmental and differentiation processes. There are five kinds of CBPs in *A. thaliana*: HAC1, HAC2, HAC4, HAC5, and HAC12 [115]. In *A. thaliana*, TGAs (TGACG-binding transcription factors) recruit the CBP family members HAC1/5 into the SA signaling cascade. This recruitment leads to the formation of a coactivator complex with NPR1 (nonexpressor of pathogenesis-related genes 1). Through histone acetylation-mediated epigenetic modifications, this complex facilitates the activation of PR (pathogenesis-related gene) transcription [131].

### 4.2. Histone Deacetylase

The HDACs in *A. thaliana* are similar to those in fungi, with Class I including HDA6, HDA7, HDA9, HDA10, HDA17, and HDA19; Class II including HDA5, HDA8, HDA14, HDA15, and HDA18; and Class III including SRT1 and SRT2. Additionally, there is an extra type of HDAC named plant-specific HD2. Plant-specific HD2 includes HD2A, HD2B, HD2C, and HD2D in *A. thaliana* [132] (Table 1).

HDA6 facilitates the acetylation of histones, suppressing the expression of PR (pathogenesis-related) genes and curtailing SA biosynthesis amid pathogen infection through direct regulation of CBP60g (Cam-binding protein 60-like g) and SARD1 (Sar deficient 1) [133,134]. Meanwhile, in *T. aestivum*, a WD40 repeat protein, TaHOS15, is discovered to recruit TaHDA6 to the chromatin [135]. This is a potential target for crop resistance improvement. HDA9 can repress the expression of NOD-like receptors (NLRs) by decreasing H3K9 acetylation and further decreasing plant immunity [136]. In *Oryza sativa*, histone deacetylase HDT701 is a member of Class I HDACs, modulating the acetylation state of histone H4. This modulation enables HDT701 to bind to the promoter areas of MAPK6 and WRKY53, thereby negatively regulating resistance to rice blast [137]. When *Ustilaginoidea virens* infects, UvSec117 is secreted into *O. sativa* and recruits HDT701 to the nucleus, decreasing H4 acetylation and the expression of resistance-related genes [138]. The resistance to bacterial blight, rice blast, and rice false smut (RFS) is adversely modulated by the histone deacetylases HDA705 (another Class I HDAC in *O. sativa*) and HDA701 [139]. As a negative regulator of plant resistance to *Pseudomonas syringae*, SRT2 is a NAD-dependent sirtuin family histone deacetylase that suppresses the genes involved in SA production, such as PAD4, EDS5, and SID2 [140].

In fact, there are also some HDACs playing positive roles in plant immunity. In *Zea mays*, histone deacetylases are repressed by histone deacetylase inhibitor HC-toxin produced by *Cochliobolus carbonum*, resulting in an immunity decrease [141]. This indicates the importance of HDACs in increasing plant immunity. HDA19 significantly bolsters black spot disease resistance by augmenting the expression of ERF1, which is related to the reaction with ethylene and activating the jasmonic acid (JA)/ethylene (ET) signaling pathway. Furthermore, HDA19 engages with transcription factors WRKY38 and WRKY62, inhibiting their transcriptional capabilities, thereby actively modulating SA synthesis and ultimately fortifying disease resilience [142,143]. In 35S:HDA19 transgenic plants, there was a notable rise in the levels of JA-regulated PR genes compared to normal plants, suggesting the potential role of HDA19 in JA-responsive mechanisms during pathogen reaction [144]. In *A. thaliana*, microbial-associated molecular patterns activate MAP kinase MAPK3; subsequently, MAPK3 binds to and phosphorylates histone deacetylase HD2B, leading to genome-wide shifts of the H3K9 acetylation landscape and immune response [145]. The HD2C of *A. thaliana* can even mediate the acetylation of *Cauliflower mosaic virus* (CaMV) histone when infected and decrease its pathogenicity. Inversely, CaMV expresses P6 protein to inhibit HD2C [146].

According to these earlier findings, HACs and HDACs mediate the acetylation and deacetylation of histones to regulate the expression of different genes and impact plant immunity.

## 5. Crosstalk between Histone Acetylation and Other Epigenetic Modifications

During the interaction between plants and pathogenic fungi, multiple post-translational modifications are often involved. The crosstalk among these modifications collaboratively regulates fungal virulence, plant resistance, and the interaction between plants and pathogenic fungi. Specifically, histone acetylation has been reported to participate in the crosstalk with multiple post-translational modifications, contributing to the interaction between plants and pathogenic fungi.

### 5.1. DNA Methylation

The interplay between histone acetylation and DNA methylation has been widely reported. In *Arabidopsis*, the histone mark H3K18ac (histone H3 lysine 18 acetylation) is a prerequisite for DNA demethylation, requiring H3K18ac deacetylation to maintain genome-wide DNA methylation status [147]. The DNA demethylase ROS1 (repressor of transcriptional gene silencing 1) plays a crucial role in plant defense against pathogens. ROS1 enhances plant resistance by demethylating the promoters of some immune-related genes, thereby enhancing their expression [8,148,149]. Moreover, it has been reported that *Arabidopsis ros1* mutants exhibit reduced resistance to pathogens [150]. Further research has revealed that ROS1 targets specific genomic regions associated with IDM1, a protein with histone acetyltransferase-like function. IDM1 reads multiple epigenetic marks (DNA methylation, unmethylated H3K4, and H3R2) and generates new epigenetic marks (histone H3K18 and H3K23 acetylation), which recruit the DNA demethylation machinery to selected genomic loci, preventing their hypermethylation and transcriptional silencing [151]. Moreover, the *Arabidopsis* histone deacetylase HDA6, as an eraser enzyme of the histone mark H3K18ac, has been reported to prevent DNA demethylation and thereby maintain the DNA methylation status in the pericentromeric region [147]. Furthermore, it has been reported that the DNA methyltransferase AtDNMT2 in *Arabidopsis* directly interacts with Type-2 histone deacetylases (AtHD2s) and participates in histone deacetylation processes [152]. In *N. crassa*, the deletion of the histone deacetylase enzyme Hda1 not only results in elevated levels of histone H3 acetylation but also leads to partial loss of genomic DNA methylation and disappearance of histone H3K9me3 [153]. These studies indicate that the crosstalk between histone acetylation and DNA methylation is not only involved in the regulation of plant resistance but also in modulating the development of pathogenic fungi.

### 5.2. Histone Methylation

Histone methylation and acetylation coordinate with each other in the regulation of chromatin structure. Acetylation is associated with chromatin loosening and gene activation, while methylation can either activate or repress gene transcription, depending on specific situations such as the methylation site and its status. The *Arabidopsis* histone deacetylase HDT1 is a transcriptional repressor of the *Arabidopsis* rRNA gene [154,155]. HDT1 has been shown to undergo deacetylation modification at the H3K9 site along rDNA chromatin, subsequently leading to dimethylation of H3K9 and inhibition of rDNA expression. During the interaction between the parasite and the host, the effector 32E03 interacts with the deacetylase HDT1 and the histone chaperone FKBP53 protein in the host plant. Moreover, the effector inhibits host plant HDAC activity and mediates the increase in H3K9 acetylation levels in rDNA coding and non-coding regions, which opens rDNA chromatin, allows increased transcription of rRNA genes, and contributes to parasitism [156]. Moreover, WRKY transcription factors in *Arabidopsis* are involved in the regulation of multiple immune-related genes. It has been reported that the acetylation of histones H3 and H4 and the methylation of H3K4 occur on the promoter of WRKY. These two modifications are jointly involved in the initiation of WRKY transcription factors during pathogen infection and salicylic acid analog treatment [157]. These studies indicate that histone methylation and acetylation coordinately participate in complex regulatory networks in plant–fungus interactions by regulating chromatin status and gene expression, affecting plant infection responses and immune mechanisms to fungi.

### 5.3. Other Epigenetic Modifications

Histone acetylation has also been reported to interplay with other epigenetic modifications, such as phosphorylation and ubiquitination. These interplays are jointly involved in the regulation of plant–pathogen interactions. The *Arabidopsis* HD2-type H3K9ac deacetylase HD2B is targeted by the MAP kinase MPK3 and plays an important role in plant resistance. In this case, MPK3 directly phosphorylates HD2B, thereby relocating it to the nucleus to regulate H3K9 acetylation levels of plant immune response-related genes [145]. The effector FolSVP1 secreted by *Fusarium oxysporum* has been reported to target the pathogenesis-related protein PR1 in the host apoplast and hijack PR1 to enter the host cell nucleus. In the host cell, the host promotes the degradation of FolSVP1 through ubiquitination at the K167 site. Nevertheless, the fungal acetyltransferase FolARD1 modifies the K167 residue of FolSVP1 through acetylation and inhibits the ubiquitination and degradation of FolSVP1, thereby ensuring the toxicity of FolSVP1 [158]. The interaction between plants and pathogenic fungi may involve a complex epidermal modification regulatory network. In addition to the above epidermal modifications, histone acetylation may also have crosstalk with other epidermal modifications. However, there are currently few relevant reports. With the advent of multi-proteomics analysis, it will be a promising research field to analyze the synergy or competition between different epigenetic modifications in the process of plant–fungi interaction.

Histone acetylation is not an independent process in organisms; it always connects with other epigenetic modifications, such as methylation, phosphorylation, and ubiquitination, indicating its important status in plant–fungi interactions.

## 6. Conclusions

Histone acetylation widely occurs in fungi and plants, and the acetylation level is dynamic in various biological processes, which are governed by different HATs and HDACs. Histone acetylation regulates gene expression by affecting the tightness between chromatin and nucleosome and then participates in biological processes, including the development and pathogenicity of pathogenic fungi, and disease resistance of plants. Many HATs and HDACs with important biological functions have been identified in fungi and plants.

Although significant progress has been achieved in understanding the role of histone acetylation in plant–fungus interaction, numerous challenges remain. For instance, further investigation is warranted to elucidate the precise regulatory mechanisms underlying protein acetylation in these interactions, as well as to explore how histone acetylation modulates plant resistance and fungal pathogenicity. Additionally, it is crucial to decipher how histone acetylation interacts with other epigenetic modifications to jointly orchestrate plant–pathogen dynamics.

Moreover, the potential applications of histone acetylation in plant–fungus interactions are vast, offering promising opportunities for the development of novel HDAC inhibitors that could bolster plant resistance or mitigate fungal virulence. As histone acetylation governs gene expression by modulating chromatin architecture, it emerges as a pivotal epigenetic modification in mediating the intricate dance between plants and fungi.

By delving deeper into the regulatory mechanisms and exploring the application prospects of protein acetylation in plant–fungus interaction, we anticipate unveiling novel insights and strategies for fine-tuning plant defense mechanisms and fungal pathogenicity.

## Figures and Tables

**Table 1:** Putative HATs and HDACs in species of fungi and plants.

Kingdom	Species	HATs				HDACs			
		HAG	HAM	HAC	HAF	Class I	Class II	Class III	HD2
Fungi	*Aspergillus nidulans*	3	6	1	1	2	2	6	0
	*Botrytis cinerea*	3	5	1	1	2	0	2	0
	*Colletotrichum graminicola*	3	5	2	1	2	2	5	0
	*Fusarium graminearum*	3	4	1	1	2	2	6	0
	*Fusarium oxysporum*	5	3	2	1	2	2	7	0
	*Magnaporthe oryzae*	4	6	0	2	2	2	5	0
	*Mycosphaerella graminicola*	3	4	0	1	2	2	9	0
	*Puccinia graminis*	4	4	1	1	5	2	3	0
	*Ustilago maydis*	3	5	0	1	4	2	4	0
Plants	*Arabidopsis thaliana*	2	18	16	2	7	7	8	2
	*Oryza sativa*	3	15	30	1	14	8	5	2

## Data Availability

Not applicable.
